# Crocin Restores Hypotensive Effect of Subchronic Administration of Diazinon in Rats

**Published:** 2013-01

**Authors:** Marjan Razavi, Hossein Hosseinzadeh, Khalil Abnous, Vahideh Sadat Motamedshariaty, Mohsen Imenshahidi

**Affiliations:** 1 School of Pharmacy, Mashhad University of Medical Sciences, Mashhad, Iran; 2 Pharmaceutical Research Center, Department of Pharmacodynamics and Toxicology, School of Pharmacy, Mashhad University of Medical Sciences, Mashhad, Iran; 3 Department of Medicinal chemistry and Dept. of Biotechnology, School of Pharmacy, Mashhad University of Medical Sciences, Mashhad, Iran; 4 Pharmaceutical Research Center, Department of Pharmacology, Mashhad University of Medical Sciences, Mashhad, Iran

**Keywords:** Crocin, Crocus sativus, Diazinon, Heart rate, Malondealdehyde, Systolic blood pressure

## Abstract

***Objective(s): ***In this study, the effects of crocin against subchronic toxicity of diazinon (DZN) on systolic blood pressure (SBP) and heart rate (HR) were evaluated in rats.

***Materials and Methods: ***Rats were equally divided into 7 groups; control (corn oil), DZN (15 mg/kg), crocin (each group received 12.5, 25 or 50 mg/kg crocin plus DZN), vitamin E (200 IU/kg plus DZN) and crocin (50 mg/kg) treated groups. Rats were given DZN via gavage once a day for 4 weeks. Vitamin E (three times per week) and crocin (once a day) were intraperitoneally injected to rats for 4 weeks. Plasma cholinesterase activity (Elman method), malondealdehyde (MDA) levels in the aortic tissue (Thiobarbituric acid reactive substances or TBARS method); SBP and HR (tail cuff method) were evaluated at the end of 4th week.

***Results: ***A significant decrease in cholinesterase activity was observed in DZN group (*P*< 0.001). Crocin did not show any effects on cholinesterase activity. DZN increased MDA levels in aortic tissue (*P*< 0.001) in comparison with control group. Crocin and vitamin E plus DZN decreased MDA elevation induced by DZN in aortic tissue. DZN significantly reduced SBP (*P*< 0.01) and increased HR (*P*< 0.001) in comparison with control. Concurrent administration of crocin and DZN, improved the reduction of SBP and the elevation of HR induced by DZN in rat. Crocin alone did not have any effect on SBP and HR.

***Conclusion: ***This study showed that concurrent administration of crocin and DZN could restore the effects of subchronic DZN administration on SBP and HR in rats.

## Introduction

Organophosphorus ester pesticides (OPs) are generally used as agricultural and domestic insecticides. OPs can damage different tissues in human and animals. Neurotoxicity, myocardial injury, cytotoxicity, respiratory failure and immune system dysfunction have been reported in OPs poisioning. The main mechanism of OPs intoxication is the inhibition of acetylcholinesterase (AChE) and overstimulation of its receptors as a result of accumulation of acetylcholine (1). However, it has been shown that cholinergic hyperexcitability may not be responsible for all of toxic effects of OP poisioning. Moreover some evidences show that other mechanisms such as novel targets and oxidative stress are involved in OPs toxicity (1-3). 

Diazinon [(DZN: O,O-diethyl-O-(2-isopropyl-4-methyl-6-pyrimidinyl) phosphorothionate)] is a commonly used thionophosphorus organophosphate (OP) pesticide. The toxicological effects of DZN in animals and human have been demonstrated in acute and chronic exposures. Changes in liver enzymes and biochemical indices and swelling of mitochondria in hepatocytes (4), toxic effects on blood cells, spleen, thymus, lymph nodes of rats (5) and on other organisms have been reported. Similarly, some studies have shown that DZN may damage the myocardium of heart in chronic exposure through oxidative stress (6). 

Crocin, is a carotenoid isolated from *Crocus sativus* L. (saffron), and is responsible for the red color of saffron. It is a pharmacologically active component of saffron. Modern pharmacological studies have demonstrated that crocin can be used as a new therapeutic agent. It has antitumor (7, 8), antioxidant, radical scavenging (9-12), hypolipaemic (13, 14) and memory-improving effects (13, 15). 

Moreover, the cardioprotective effects of saffron and its active components such as crocetin and crocin have been reported in some studies that are related to modulation of endogenous antioxidant enzymatic activities (16). 

OPs such as paraoxone and dichlorvos attenuate arterial smooth muscle contraction in rats (1). Several studies reported that acute and chronic OPs toxicity could damage the vascular wall and lead to degeneration of collagenous and elastic fibers (17-19), there are also few reports on chronic cardiac toxicity of DZN and other Ops. Thus this study was conducted to evaluate the effects of subchronic intoxication of DZN on blood pressure (BP) and heart rate (HR) in rats and also to assess the possible protective effects of crocin as an antioxidant agent on these parameters.

## Materials and Methods

***Chemicals***

DZN (Bazodin®, Cyngenta, purity 96 %) and vitamin E were purchased from OSVE Pharmaceutical Co. (Tehran, Iran). TBA (2-thiobarbituric acid), n-butanol, phosphoric acid, potassium chloride and MDA were obtained from Merck. Stigmas of *C. sativus* L. obtained from Novin Saffron (collected from Ghaen, Khorasan province, Northeast of Iran) was analyzed in accordance to the ISO/TS 3632-2. Crocin was extracted and purified as defined by Hadizadeh and colleagues (20). 

***Animals and treatment***

Adult male Wistar rats (weight 200–250 g) were provided by Animal Center (School of Pharmacy, Mashhad University of Medical Sciences). They were maintained on a 12 hr light/dark cycle and at a temperature of 23±1 ^◦^C with free access to food and water. These conditions were kept constant throughout the experiments. All animal experiments were carried out in accordance with Ethical Committee Acts of Mashhad University of Medical Sciences.

The rats were randomly divided into seven groups: 1) control group (corn oil); 2) DZN treated group (15 mg/kg); 3) DZN + crocin 12.5 mg/kg treated group; 4) DZN + crocin 25 mg/kg treated group; 5) DZN + crocin 50 mg/kg treated group; 6) DZN + Vit E 200 IU/kg treated group and 7) Crocin 50 mg/kg group. All groups consisted of six rats. DZN was treated via gavage once a day for 4 weeks. Corn oil (vehicle of DZN) was given in the same way to control rats. Vitamin E and crocin were intraperitoneally administrated for 4 weeks three times a week, once a day, respectively.

***Measurement of body weight***

The body weights of control and treated rats were measured at the beginning and end of the 4th week.

***Determination of cholinesterase activity***

After 4 weeks of treatment, rats were killed and sera were collected. The activities of cholinesterase in plasma were analysed using Ellman method. This method is based on the hydrolysis of acetylthiocholine iodide. The reaction of the thoil compound with 5,5'-Dithio-bis (2-nitrobenzoic acid) (DTNB) produces a color-forming compound with absorbance at 405 nm. 20 µl of sample serum and 100µl of 5% (w/v) acetylthiocholine iodide solution were added to 3 ml Elman reagent (0.02% DTNB in 0.1M sodium dihydrogen phosphate buffer solution, pH=7.4). The absorbances of different samples were recorded at 0.5 min intervals for 2 min.

 The cholinesterase activity was calculated as below:

Cholinesterase (mU/ml, at 25 ºC) = change in absorbance in 30 sec.×23400 (21).

***Measurement of malondialdehyde***

For measurement of MDA (as an important marker of oxidative stress), the thoracic aorta of rats in different groups were removed after 4 weeks treatment and were washed with normal saline. They were homogenised at 4 °C (Polytron, Kinematica, Switzerland) in the KCl 1.15% in order to produce a 10% homogenate. These homogenates were centrifuged at 6000×g for 10 min to obtain supernatants. The levels of protein and MDA were measured in the supernatants (22). The protein content of homogenates was determined by Bio-Rad Protein Assay Kit according to the kit protocol.

 MDA levels were determined according to a method of Fernandez *et al* (23). The principle of the method is spectrophotometric measurement of the color developed during reaction to thiobarbituric acid (TBA) with MDA. To this end, 3 ml phosphoric acid (1%) and 1ml TBA (0.6%) were added to 0.5 ml of supernatant in a centrifuge tube and the mixture was heated for 45 min in a boiling water bath. After cooling, 4 ml of n-butanol was added to the mixture and vortex-mixed for 1 min followed by centrifugation at 2,000 g for 20 min. The organic layer was transferred to a fresh tube and its absorbance level was measured at 532 nm.

The concentration of MDA in the arotic tissue was calculated by the standard curve of MDA and expressed in nmol/mg of protein (22).

***Measurement of systolic BP and HR***

Three days before the last treatment, the training of rats for indirect BP measurements was started. This training consisted of the regular handling of the animals and getting used to the restraining cage and the tail-cuff. SBP was measured by a tail cuff method the day after 4 weeks of treatment in all groups as described by Lorenz (24). Briefly, rats were heated for approximately 15 min at 30-32 °C to increase blood flow to the tail. After that, animals were placed in small restraining cages with a cuff around the proximal end of the tail. After placing the cuff, a pulse transducer was used near the end of the tail. Then the tail cuff was inflated using the related botton on the NIBP (Non-Invasive Blood Pressure) controller apparatus and SBP pressure and HR were calculated from the lab chart software. The mean values of 5 BPs and HRs readings were used for each animal.

***Statistical analysis***

All data were presented as mean±SEM. The statistical comparisons among groups in each experiment were done with one-way analysis of variance (ANOVA) followed by Tukey-Kramer test for multiple comparison. *P* values less than 0.05 were considered significant. 

**Table 1:** Effects of DZN and crocin treatment on body weight changes after 4 weeks treatment

Groups	Alteration in body weight (g) (mean ±SEM)
Control	29.98 ± 2.94
DZN 15 mg/kg	12.13± 0.97***
Crocin 50 mg/kg	25.12 ± 1.85
DZN+Vitamin E (200mg/kg)	18.09 ± 2.73**
DZN+Crocin 12.5 mg/kg	15.82 ± 1.44***
DZN+Crocin 25 mg/kg	13.32 ± 2.09***
DZN+Crocin 50 mg/kg	14.02 ± 1.004***

Vitamin E and crocin were administered intraperitoneally, three times per week, once a day, respectively. DZN (corn oil as a vehicle) was treated via gavage to rats once a day. Data are shown as mean ± SEM, ***P< 0.001 and**P< 0.01 compared to the control group. One way ANOVA, Tukey-Kramer, n= 6

## Results

***Effect of*** ***DZN*** ***and crocin on rat body weight***

Body weight significantly decreased in DZN treated group after 4 weeks in comparison with the control group (*P*< 0.001). No significant differences were observed in body weights of crocin 50 mg/kg and control group after 4 weeks of treatment. Also no significant changes were observed in body weights in crocin+ DZN (three doses) and vit E + DZN treated groups after 4 weeks of treatment in comparison with the DZN group (Table 1).

***Effect of DZN and crocin on plasma cholinesterase activity***

A significant decrease was observed in plasma cholinesterase activity in the DZN treated group in comparison with the control group (*P*< 0.001), but no significant difference was observed between DZN plus crocin groups (all three doses) or Vitamine E and DZN treated groups ().

***Effect of DZN and crocin on MDA level in aortic tissue***

**Figure 1:**
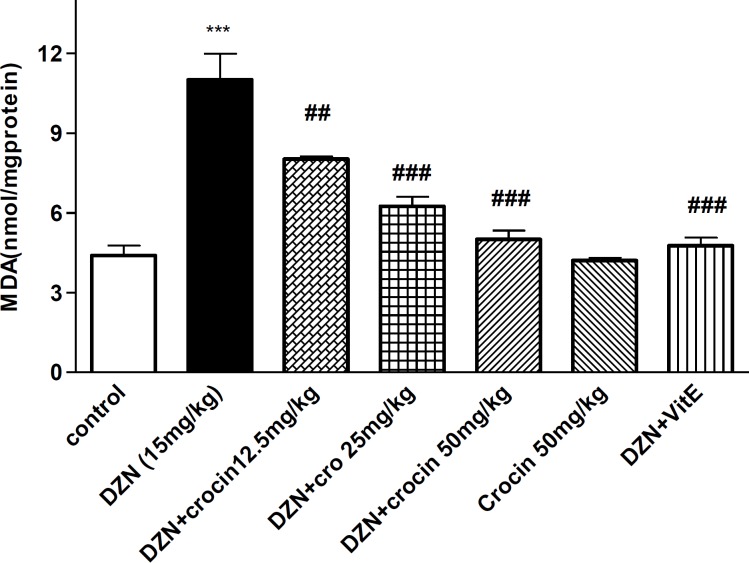
Effects of DZN and crocin treatment (4 weeks) on MDA Level in the aortic rat tissues. Vitamin E and crocin were administered intraperitoneally three times per week, once a day, respectively. DZN (corn oil as a vehicle) was given through gavage to rats once a day. Data are shown as mean± SEM, ***P< 0.001 compared to the control group, ^###^*P*< 0.001 and ^##^*P*< 0.01 compared to DZN group, One way Anova, Tukey-Kramer test, n=6

The treatment by DZN significantly increased MDA level in thoracic aorta in comparison with the control (*P*< 0.001), crocin (three doses) and vitamin E decreased MDA level in concurrent administration with DZN (*P*< 0.001, *P*< 0.001) (Figure 1).

***Effect of DZN and crocin on SBP***

**Figure 2:**
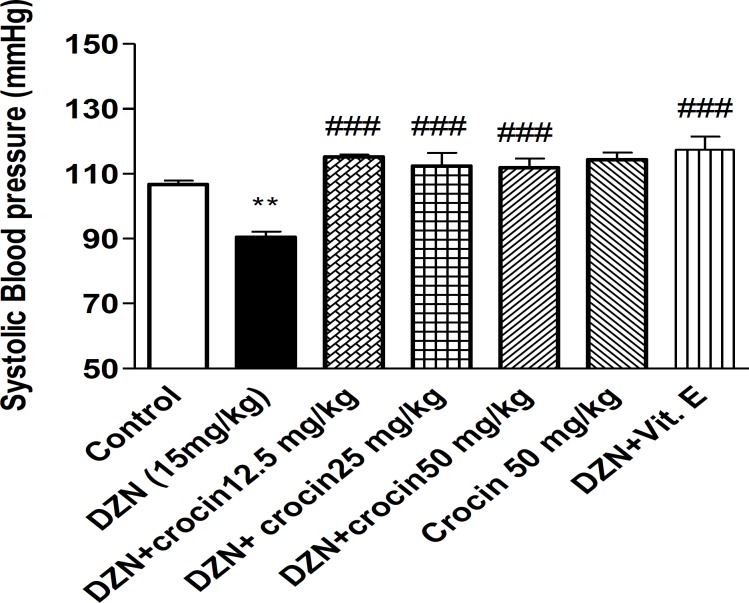
Effects of DZN and crocin treatment (4 weeks) on SBP in rats using tail cuff method. Vitamin E and crocin were administered intraperitoneally three times per week, once a day, respectively. DZN (corn oil as a vehicle) was given through gavage to rats once a day. Data are shown as mean ± SEM of five BP readings, **P< 0.01 compared to the control group, ^###^*P*< 0.001 compared to DZN group, One way ANOVA, Tukey Kramer test, n= 6

As shown in Figure 2, a significant decrease was observerd in SBP in DZN treated group in comparison with the control (*P*< 0.01). There was no significant difference between crocin (50 mg/kg) and control. A significant increase was observed in crocin (three doses) plus DZN and vitamin E plus DZN in comparison with DZN groups (*P*< 0.001). 

**Table 2:** Effects of DZN and crocin treatment (4 weeks) on plasma cholinesterase activity in rats

Groups	Cholinesterase activity (mU/ml) (mean ±SEM)
Control	679.56±11.93
DZN 15 mg/kg	256.56± 15.02***
Crocin 50 mg/kg	659.56 ± 10.78
DZN + Vitamin E	279.33 ± 7.31***
DZN + Crocin 12.5 mg/kg	302.72 ± 8.73***
DZN + Crocin 25 mg/kg	277.29 ± 6.02***
DZN + Crocin 50 mg/kg	269.1 ± 10.94***

Vitamin E and crocin were administered intraperitoneally three times per week, once a day, respectively. DZN (corn oil as a vehicle) was given through gavage to rats once a day. Data are shown as mean ± SEM, ***P< 0.001 compared to the control group, One way ANOVA, Tukey-Kramer test, n= 6

***Effect of DZN and crocin on HR***

**Figure 3:**
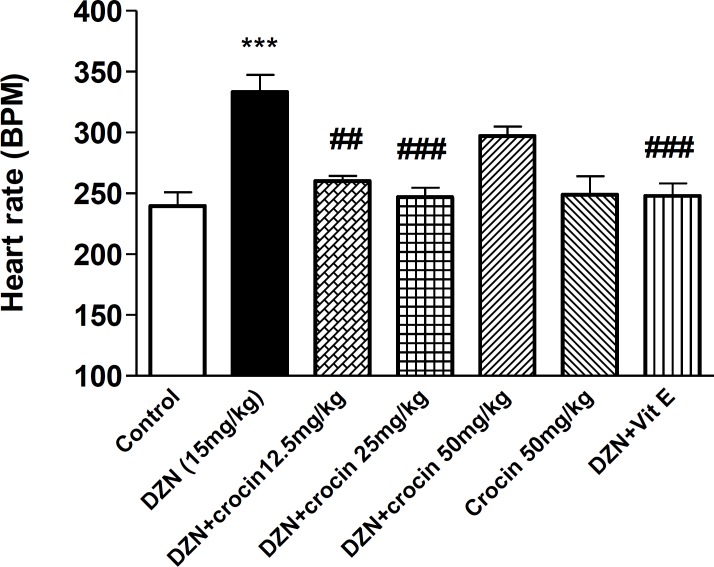
Effects of DZN and crocin treatment (4 weeks) on heart rate (HR) in rats using tail cuff method. Crocin and vitamin E were administered intraperitoneally three times per week, once a day, respectively. DZN (corn oil as a vehicle) was given through gavage to rats once a day. Data are shown as mean ± SEM of five HR recording, ***P< 0.001 compared to the control group, ^###^*P*< 0.001 and ^##^*P*< 0.01 compared to DZN group, One way ANOVA, Tukey-Kramer test, n= 6

As shown in Figure 3, a significant increase was observerd in HR in DZN treated group in comparison with the control (*P*< 0.001). There was no significant difference between crocin (50 mg/kg) and control groups. A significant decrease was observed in crocin (12.5 and 25 mg/kg) plus DZN and vitamin E plus DZN in comparison with DZN groups (*P*< 0.01, *P*< 0.001). 744

## Discussion

In this study, after 4 weeks of DZN administration, a reduction in plasma cholinesterase activity was observed in comparison with the control group. The concurrent administration of DZN and crocin or vitamin E did not affect the cholinesterase activity. Thus, it may be concluded that crocin does not have any effect on cholinesterase inhibition of DZN.

Also, loss of body weight was observed 4 weeks after DZN-treatment. DZN is known to show its toxic effects by inhibiting cholinesterase activity which could be due to the less food consumption and/or fluid and electrolyte loss, as a result of a reduction in cholinesterase activity induced by DZN. Crocin and vitamin E did not prevent the reduction of body weight changes induced by DZN after 4 weeks. As crocin or vitamin E did not have any significant effect on cholinesterase activity, so it can be postulated that a reduction in body weight changes in treated groups is a result of cholinesterase inhibition of DZN (25). 

It has been proved that OPs chronic toxic effects have been accompanied with oxidative stress damages in different organs such as cardiovascular system (6, 26-29). 

Malondialdehyde, the final product of lipid peroxidation, resulting from the interaction of reactive oxygen species and the cellular membrane, has been considered to evaluate the presence of free radicals and lipid peroxidation induced cardiovascular toxicity (6, 29). In this experiment, DZN caused a significant increase in MDA level in the aortic tissue after 4 weeks treatment. 

This study demonstrated a decrease of SBP and an increase of HR in rats treated by DZN (15 mg/kg) for 4 weeks in comparison to the control group. The concurrent administration of DZN and crocin (three doses) could restore hypotensive effect of DZN. Also, tachycardia induced by DZN, restored nearly to the level of control group by concurrent administration of DZN and crocin (12.5 and 25 mg/kg). The effect of crocin on both BP and HR at the highest dose (50 mg/kg) was similar to the control group. 

As a significant increase in the MDA level was observed in the aortic tissue, therefore, it could be suggested that elevated products of stress oxidative such as reactive oxygen species (ROS) during DZN administration, caused alterations in vascular system, so that tonicity and elasticity of vascular smooth muscle cells can be affected by DZN (29). Thus, one of the mechanisms involved in hypotensive effect of subchronic treatment of DZN, may be related to the disturbances in vessel smooth muscle cells, particularly microvessels. Macrovascular dysfunction induced by OPs has been reported previously. It has been shown that acute i.v. administration of OPs such as DZN and fenthion, lead to hypotensive effect and inhibited contraction induced by KCl and phenylephrine in isolated rat aorta, through direct inhibitory effect on vessel smooth muscle cells which is a result of reduction of intracellular calcium concentration (30), but there is no evidence proving that the microvascular dysfunction has been induced by OPs in chronic toxicity.

Considering down regulation of muscarinic cholinergic receptors during chronic toxicity of OPs (31), it can be suggested that DZN administration after 4 weeks may cause reduction in muscarinic cholinergic receptors, therefore one of the mechanisms involved in tachycardia induced by DZN, may be due to the down regulation of receptors during DZN treatment. In this study, the day after discontinuing of DZN after 4 weeks, HR was measured and tachycardia was observed.

The reflex tachycardia in response to hypotension induced by DZN appears to be another mechanism involved in HR elevation of DZN through baroreceptor reflex mechanism.

Similarly, it seems that the increase of nitric oxide level by subchronic DZN exposure may be considered as another mechanism involved in DZN vascular toxicity (32).

 Crocin, one of the pharmacological ingredients of saffron, has a potent antioxidant activity (14). The protective effect of saffron and its constituent, crocin, has been shown in previous studies. It was demonstrated that saffron and its constituent, crocin have a protective effect on rats’ myocaridial injury induced by isoproterenol through modulation of oxidative stress (16, 33, 34).

In this study, the concurrent administration of crocin and DZN could improve lipid peroxidation induced by DZN in aortic tissue. This result showed that crocin protected cardiovascular damages induced by DZN as a result of oxidative stress through scavenging free radicals.

Also, in this experiment crocin plus DZN could restore the decrease of SBP and the increase of HR after 4 weeks. Although crocin could reduce the elevation of HR induced by DZN at doses of 12.5 and 25 mg/kg plus DZN through antioxidant and scavenging of free radical activities, this effect was not significant at crocin 50 mg/kg plus DZN. It seems that the elevation of HR by crocin at the highest dose may be related to its effect on adenylate cyclase, ardenorecptors or intracellular calcium. 

Vasodilatory effect of acute administration of crocin was demonstrated in Imenshahidi *et al* study (35) and in another study, it has been mentioned that aqueous-ethanol extract of *C. sativus* has a potent inhibitory effect on heart rate and contractility of guinea pig heart (36), also relatively potent stimulatory effect of *Crocus sativus* extract on β_2 _adrenoceptors has been reported previously (37), However, in this study, we found a different effect from crocin. For explanation of this result, it can be postulated that concurrent administration of crocin and DZN during 4 weeks, plays a modulatory role in alterations caused by DZN in the rat cardiovascular system through scavenging of free radicals and antioxidant activity, therefore crocin could restore the reduction in BP induced by DZN. 

## Conclusions

This study showed that the concurrent administration of crocin and DZN could restore the subchronic effects of DZN on SBP and HR in rats, that was partly due to the antioxidant properties of crocin by decreasing the lipid peroxidation in the rat cardiovascular system.
